# The First Pint of Science Festival in Asia

**DOI:** 10.1177/1075547017739907

**Published:** 2017-11-03

**Authors:** Matthew T. Robinson, Nattapat Jatupornpimol, Sandy Sachaphimukh, Maria Lönnkvist, Andrea Ruecker, Phaik Yeong Cheah

**Affiliations:** 1 Laos-Oxford-Mahosot Hospital-Wellcome Trust Research Unit, Mahosot Hospital, Vientiane, Lao PDR; 2 University of Oxford, Oxford, UK; 3 Mahidol-Oxford Tropical Medicine Research Unit, Mahidol University, Bangkok, Thailand

**Keywords:** public engagement, Pint of Science, Thailand, Asia, science festival

## Abstract

The Pint of Science Festival is the largest annual international science festival. So far the event has been held simultaneously in Europe, North America, South America, Africa, and Australia but not in Asia. Pint of Science Thailand was held for the first time this year, in Thailand’s capital, Bangkok. This article briefly discusses some of the successes, challenges, and lessons learnt associated with running the first Pint of Science event in Asia, a culture very different to the Western Hemisphere cities that have currently hosted Pint of Science events.

## Introduction

The Pint of Science Festival is the biggest annual international science festival (Paul & Motskin, 2016). In 2016 it was held simultaneously in over 100 cities, in 12 countries, across five continents (Paul & Motskin, 2016). The festival, created by Drs. Michael Motskin and Praveen Paul, began in 2013 and was first held in 15 pubs across three cities in the United Kingdom (London, Oxford, and Cambridge). Since then the festival has expanded across Europe, North America, South America, Africa, and Australia, but as yet no festival has been held in Asia (or Antarctica). The aim of the Pint of Science Festival is to provide a relaxed and enjoyable environment where members of the general public, who may have an interest in science but might have little or no formal scientific training, could meet and interact directly with scientists. Researchers across all STEM (science, technology, engineering, and mathematics) subjects, from all academic levels (PhD students, postdoctoral researchers, and PIs) give fun, interesting, and interactive talks about their current scientific research, while audience members get the opportunity to ask questions, discuss topics, and hopefully leave the event with a greater understanding of science and today’s research world. The event is generally targeted to ages 18 and above, although this varies between countries and locations depending on where the events are held.

In Thailand, science engagement events are few and far between and are generally limited to schools and museums. The Mahidol-Oxford Tropical Medicine Research Unit (MORU), a Wellcome-funded research unit in Bangkok, Thailand, has an active public engagement group. Recent activities include holding Café Scientific events in both Bangkok and Vientiane (in Lao PDR; Cheah, Newton, & Mayxay, 2016), and a Village Drama Against Malaria program (Lim, Peto, Tripura, & Cheah, 2016). The Pint of Science Festival was seen as being a potentially highly successful mechanism for increasing public engagement in Thailand and other neighboring Asian countries. Therefore, in May 2017, the first Pint of Science Festival to be held in Asia took place in Bangkok, Thailand.

## Organization of the Event

Organizing for Pint of Science Thailand (PoST) started in January 2017, with the event taking place from the May 15 to 17, concurrently with other Pint of Science events around the world. The event is held under direction of the main Pint of Science management team based in the United Kingdom, and permissions were obtained, which allowed use of the name, branding, and website, as well as access to support and resources such as images, predesigned posters, and marketing material. The Thailand organizing committee consisted of a Country Director (MTR) who liaised with the main Pint of Science management group and maintained the website; a Co-Director (PYC) who oversaw permissions and funding for the event; an Event Manager (NJ) who organized the venue, equipment, posters, and speakers and was assisted by two Assistant Event Managers (AR and ML); and a Social Media Manager (SS) who oversaw the Twitter and Facebook accounts, and advertising. All committee members were actively involved in the planning and organization of the event.

## Event Location

Pint of Science is held at multiple venues in several cities throughout a hosting country. As this was a new venture in Thailand (and Asia), and there was no indication of how the event would be received, it was decided to focus on a single venue over the three consecutive festival nights. In the United Kingdon there is a culture of socializing in pubs and bars, and Pint of Science events are held in these locations as a way to provide a familiar, relaxed, and close-knit atmosphere. Although bars are found throughout Bangkok, there is no similar pub or bar culture as in the United Kingdom or Western Europe, so the decision was made to hold the event at a café in Ari—a lively and active residential neighborhood, close to the center of Bangkok, and easily accessible by the local transport system. The café was already known for its involvement in technology and creative events and as a space for people to meet and share ideas, and so the staff and management were able to assist in the organization of the festival. Additionally the venue was able to provide a variety of refreshments for the event (both food and drink).

## Speakers and Talks

The event format was to follow the standard Pint of Science Festival program, with three event nights, each consisting of three different talks from local researchers. In total, nine talks were selected for the event (Table 1). Speakers were invited from collaborating institutions to ensure diversity of topics; subject areas covered included genetics, antimicrobial resistance, modelling disease epidemics, diagnosis of infections, and rock art of South East Asia. All talks had accompanying slide presentations, and one talk (focusing on epidemics) revolved around an interactive game where participants modeled a disease outbreak. The event was planned so that one talk per night would be in Thai, while the other two talks would be in English. All the speakers offered their time voluntarily, although a small reimbursement was offered for transport to and from the venue. The standard Pint of Science format has specific topic areas each night (Beautiful Mind, Atoms to Galaxies, Our Body, Planet Earth, Tech Me Out, or Our Society), usually with different venues hosting different topics. Because only one venue was being used, and the majority of selected talks focused on biological research, we avoided these general themes and gave each night an overall title (Table 1).

**Table 1.** Topics and Talks for Each Event Night.

Event night	Talk title	General topic
1. Killer Bugs: Disease Detection and Destruction		
	Applied Proteomics: A Short Story of Cake and Urine	Diagnosis of pathogen infections
	Bac Chat	Genetics of bacteria
	Antibiotic Footprint in Thailand	Antibiotic use
2. The Hidden Secrets of Evolution and Epidemics		
	Crouching Tiger, Hidden Elephants: The Unseen Cave Paintings of Southeast Asia	Rock art in Southeast Asia
	Time Machines and the Modeling Game	Modelling epidemics
	Evolution Director	Evolution of enzymes
3. Tackling Diseases of the Past and Present		
	Precision Medicine in Cancer: How to Make Your Gene Talk	Personalized medicine
	Leprosy: Discovered by a Norwegian, Still Causing Problems Today	Leprosy
	The Sex Lives of Malaria Parasites	Malaria cell biology and malaria control

## Pint of Science Thailand Website

The PoST website (www.pintofscienceth.com) was set up using templates provided by the Pint of Science management group. The website provided information and brief history of the event, details of each event night (including the talks and presenters for each night), location of the event, and a hyperlink to reserve tickets for each night. Website administration was carried out by the organizing committee and was a focal point to provide information to interested parties. The website was linked directly to the main Pint of Science website (www.pintofscience.com). Specific areas of the website were displayed in both English and Thai, to make it widely accessible.

## Admission and Ticketing

Although the majority of Pint of Science events charge a small admission fee to cover venue charges (e.g., £4.00/€2.00), it was decided that the Thailand festival was to be free, with the aim that more people from a wider background may be encouraged to attend the event. We also provided snacks and a soft drink per attendee free of charge. Tickets were issued through Eventbrite, a free event management website for organizers (www.eventbrite.com), with 70 tickets per night made available. Interested attendees were able to click-through directly to ticket reservation from the PoST website or could discover the event by searching on Eventbrite. We asked all attendees to register on arrival, allowing us to capture some basic attendee data for later analysis (e.g., how they found out about the event and if they were associated with any particular organization).

## Advertising and Branding

Due to the short period of time for planning the event, and that a Pint of Science Festival had not been in Thailand before, advertising the event was important. Advertising was done through popular social media channels in Thailand (Facebook, Line, and Twitter), websites (www.pintofscienceth.com, www.eventbrite.com and www.tropmedres.ac), e-mail lists, and posters. To maximize our reach the event was actively advertised on Facebook.

The Pint of Science Festival has specific branding and logo (a pint glass containing beer with a brain wearing spectacles on top). Following discussions with Thai colleagues who pointed out that the direct translation of Pint of Science into Thai has no connotation, and because of cultural differences such as a food—rather than drinking—culture in Thailand, it was decided to translate Pint of Science to วิทยาศาสตร์จานหนึ่ง (“A Plate of Science”), which was felt to be more applicable culturally. No change was made to the logo or English name in order to maintain some level of connective branding.

## Outcomes From the Event

### Attendance

**Figure 1.**
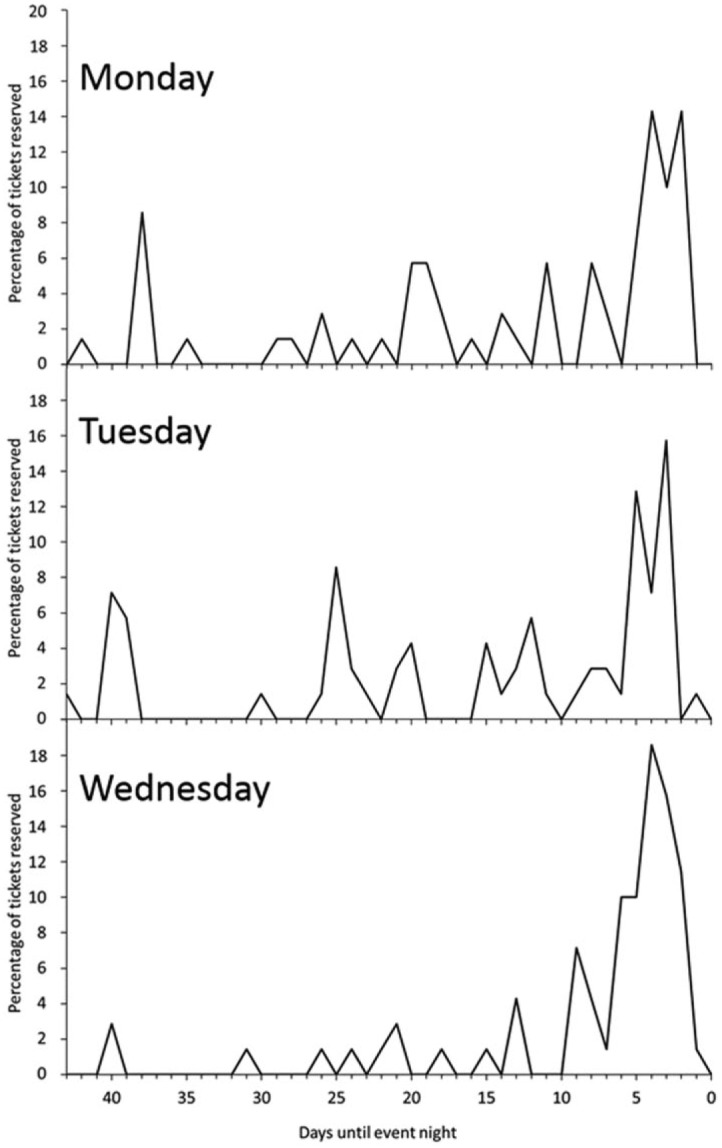
Percentage of tickets reserved by day on Eventbrite. Day 0 is the event night.

Out of 210 free tickets made available through Eventbrite, 209 were reserved in total. All tickets were allotted for both Monday and Wednesday events whilst one ticket remained for Tuesday night. Peak ticket booking period was between 2 and 6 days before each event night, representing 50.2% of all ticket reservations (Figure 1). Culturally, it is accepted for people to turn up at an event without prebooking a ticket, even more so if tickets are free. To that effect, despite almost all the tickets being reserved, the precise number of attendees could not be predicted. Overall, attendance for the event was beyond expectation, with 149 attendees over the three nights (Table 2). The majority of attendees did not prebook tickets, with 57.7% of attendees not having a ticket. Overall, 41.1% of those who reserved tickets actually attended the event. On the Tuesday event, 28% of attendees had also attended the first night, while on the Wednesday 40% of attendees had attended at least one previous night (Monday and/or Tuesday). Out of 115 individuals that attended the event, 9.6% attended all 3 nights.

**Table 2.** Attendance for the First Pint of Science Festival in Thailand.

	Monday	Tuesday	Wednesday	Total
Ticketing				
Available tickets	70	70	70	210
Tickets reserved	70	69	70	209
Attendees				
Total	53	53	43	149
With ticket	32	28	26	86
On door	21	25	17	63
Demographic (% of attendees Thai nationals)^a^				
Expected (%)	50.0	49.3	37.1	45.5
Actual (%)	58.5	56.6	44.2	53.7

a Demographic data are the proportion of audience of Thai origin, based on registration information from Eventbrite ticket reservations (expected data) or on-door registration (actual data).

### Advertising

It is difficult to determine the impact various social media strategies and advertising campaigns had. The Facebook campaign reached over 74,500 Facebook users in total (the number of unique users who saw the advertisement) and made over 86,500 impressions (number of times the advert was potentially seen by someone). This resulted in 1,241 visits to the event pages (link clicks) and 742 “likes” (where users show support for a specific item). The Eventbrite listings received a total of 347 impressions which resulted in 16.7% of all ticket reservations. The top three ways that the public interacted with Eventbrite and discovered the PoST event (accounting for 87.6% of impressions) were through sharing on Facebook from Eventbrite, by directly searching for events on Eventbrite, and from promotions of upcoming events on Eventbrite and other ticket orders.

Data captured from the on-site registration suggested that the majority of attendees heard about the events through contacts and internal advertising at MORU (23%), colleagues and friends (21%), Facebook (15%), and Eventbrite (8%).

### Attending Demography

The demography of the audience was very difficult to gauge before the event. The aim was to attract Thai locals rather than expat community. Based on information captured on Eventbrite the expected demographic across the three nights was 45.5% Thai-origin. Information captured via on-door registration indicated that actual demography was higher, with 53.7% of people attending being of Thai origin (Table 2).

### Event Language

The intention was the event to be dual language with at least one talk per night in Thai with hosting done in both Thai and English. On the first night, after establishing the demography of the audience, it was decided that the hosting would be in English. The Thai-language presenter for the first night also decided to give the talk in English rather than Thai. On the second night, the speaker gave the talk in Thai but used English-language version slides. For the third night the speaker presented in English. This speaker raised an important point: much of his work was carried out in English as the technical and scientific terms he used had no direct Thai translation.

The difference between the Thai name for the event and the English name and logo caused more confusion than anticipated, after receiving feedback from a number of people who attended the event (questions such as why the names were different in Thai and English). Maintaining the original name or translating the name to a more drink-related description may have been more preferable.

### Feedback

**Figure 2.**
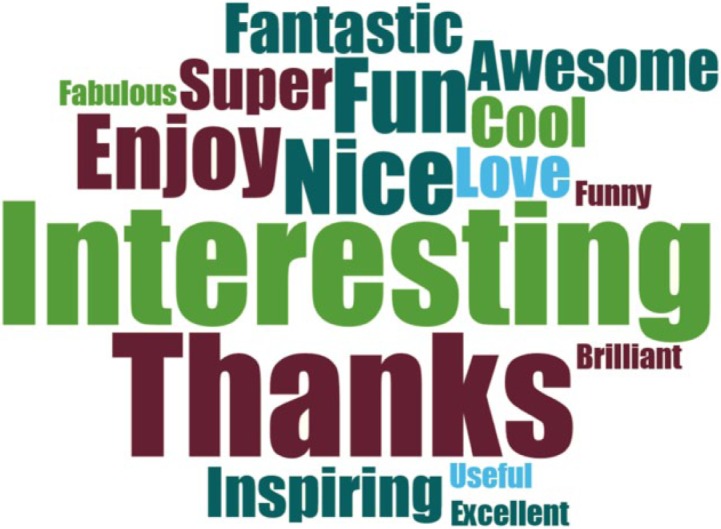
Keywords from “What’s cool?” comments, with frequency depicted by size of word.

To measure the success of PoST we asked attendees to provide feedback in the form of comments answering the question, “What’s cool? What’s not so cool?” via sticky notes placed on a large board. During the event we received 71 comments, 70% of which were classed as “cool” (Figure 2), the remaining 30% of comments were mainly suggestions for improvements rather than specifically “not cool” comments. These suggestions were very constructive and covered areas such as venue size (too small), audiovisual (difficult to hear the speaker sometimes and screen being too small), timing of the evening (longer talks, more social time afterwards), and talk contents (too complex or too easy). Many commented that they enjoyed talks that included an activity or game.

Sessions were interactive and engaging, with audience members asking many questions. We received positive casual verbal feedback from speakers and audience members stating that they found the event useful and enjoyable (“It’s actually putting things from numbers to simple concepts that people can really understand” PoST audience member) and would recommend friends to attend future PoST events.

### Postevent Engagement

Throughout the event a photographer/videographer was present to record the event. As well as still shots, which will be used for publicizing future events, an introduction video for the event was produced (YouTube, 2017), as well as recordings of each talk. These videos are now available to the public on YouTube (search for PoST) and the PoST and MORU websites.

## Conclusions

The event exceeded the expectations of the organizers, speakers, and supporters. Feedback was overwhelmingly positive, with the negative comments being only minor issues that could be easily corrected for any future event. A key issue was the size of the venue, and certainly the interest around the Pint of Science event would suggest larger venues could be used in the future. Typically, the Pint of Science Festival format is organized into one of six specific topic areas each night, with different venues hosting different topics. Although this model was not used this time, due to the limitations this would have caused with grouping talks together, future events in Thailand could use this model. In turn, this may allow small and more intimate venues to be used as attendees would likely choose specific topics that they would be interested in, rather than attending all topics at a single venue. Similarly, with less than half of those who reserved tickets attending, it was very difficult to determine expected audience size. In future, a small charge for tickets would be considered to encourage those who purchase tickets to attend; this would also make planning for the venue easier. Charging a small fee has been adopted throughout all Pint of Science events internationally, and has seen improved attendance and ability to plan (M. Motskin, personal communication, 2017). Although this still does not discount those attendees that are likely to turn up at the event without tickets.

The demography of those who attended also exceeded expectations although more targeted advertising could be planned for future events. Now that people are more aware of the event, one possible way to encourage more Thai people to attend is having specific Thai-language event nights (e.g., 1 or 2 of the 3 nights in Thai language) or locations (all talks at one location in Thai), rather than dual-language sessions.

The reception received by PoST is very encouraging for public engagement in Thailand. There is scope for the event to be expanded, not only with more venues in Bangkok but also across Thailand, and other Asian countries.
